# Supplementary material for: Transcranial Recording of Electrophysiological Neural Activity in the Rodent Brain in vivo Using Functional Photoacoustic Imaging of Near-Infrared Voltage-Sensitive Dye
Source: Front Neurosci. 2019 Aug 9;13:579. doi: 10.3389/fnins.2019.00579 (PMC6696882; doi:10.3389/fnins.2019.00579)
Supplement: Supplementary Figure S1 — PA imaging characterization. (a) spatial full-width-half-maximum (FWHM) beam profile in axial and lateral directions at various depth-of-interest (i.e., 20, 25, and 30 mm). (b) Fractional change in energy fluctuation with various number of frame averaging (i.e., 1, 5, 10, 20, 40 frames). (c) Long-term comparison in laser energy fluctuation between 0–2 min to 8–10 min time ranges. (d) Normalized PA intensity for 8 min. PA intensity averaged for 1–2 min was used as a reference intensity. White dotted rectangular in the PA image of seizure control rat indicates the photo-bleaching ROIs. [file Data_Sheet_1.docx]

Supplementary Material

Transcranial recording of electrophysiological neural activity in the rodent brain *in vivo* using functional photoacoustic imaging of near-infrared voltage-sensitive dye

Jeeun Kang^1,2^, Haichong K. Zhang^1,2^, Shilpa D. Kadam^3^, Julie Fedorko^2^, Heather Valentine^2^, Adarsha P. Malla^4^, Ping Yan^5^, Maged M. Harraz^4^, Jin U. Kang^1^, Arman Rahmim^2^, Albert Gjedde^2,6^, Leslie M. Loew^5^, Dean F. Wong^2,4,6,7,8^, Emad M. Boctor^1,2 *^

*** Correspondence:** Dr. Emad M. Boctor: [eboctor1@jhmi.edu](mailto:eboctor1@jhmi.edu)

# Characterization of fPA imaging performance.

The performance of the PA imaging system was evaluated. The wire target with 150-μm diameter was placed in the water tank, and the PA data were obtained at 20-, 25-, and 30-mm distance which are in the depth-of-interest for our lipid vesicle and *in vivo* experiments. The PA image sequence was reconstructed by using a delay-and-sum beamforming, and envelope was detected for the bandwidth 1 - 5MHz.


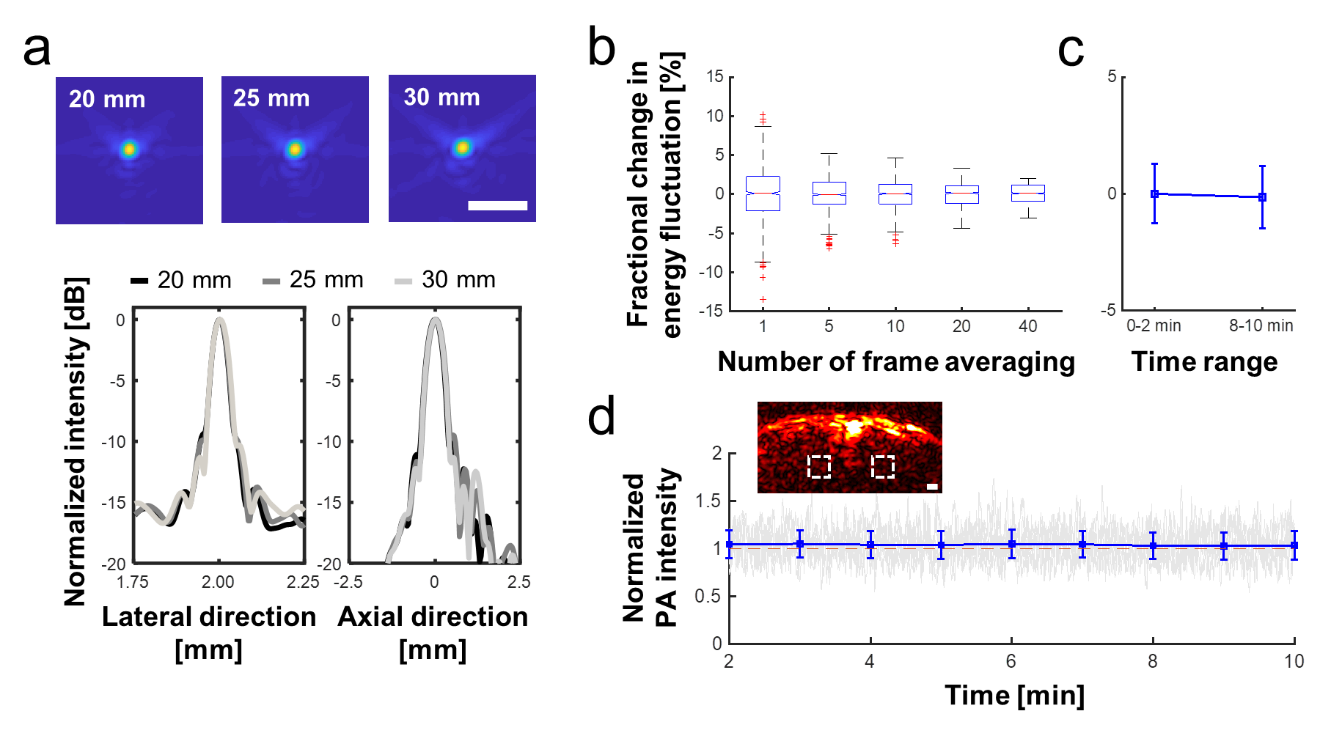


Fig. S1. PA imaging characterization. (a) spatial full-width-half-maximum (FWHM) beam profile in axial and lateral directions at various depth-of-interest (i.e., 20, 25, and 30 mm). (b) Fractional change in energy fluctuation with various number of frame averaging (i.e., 1, 5, 10, 20, 40 frames). (c) Long-term comparison in laser energy fluctuation between 0-2 min to 8-10 min time ranges. (d) Normalized PA intensity for 8 min. PA intensity averaged for 1-2 min was used as a reference intensity. White dotted rectangular in the PA image of seizure control rat indicates the photo-bleaching ROIs.

The center frequency and fractional bandwidth of the ultrasound linear array transducer are 6.88 MHz and 78.25% at -6dB level, covering the imaging bandwidth used in this study. Moreover, bandwidth below -6dB level would still be detectable. The designated imaging bandwidth is essential to have (1) deep imaging sensitivity by alleviating acoustic attenuation in skull and scalp layers and (2) better sensitivity for the PA signal bandwidth generated from tissue-scale VSD redistribution mechanism, rather than from blood contents in neurovasculatures with diameter from 10-μm to 200-μm in rat brain (Zhang, et al., 2015). It is well known that PA signal bandwidth would be inversely proportional to absorber size when thermal/stress confinements are met (Sun and Gerstman, 1999). Our imaging bandwidth extended from 1MHz to 5Hz is optimal for sphere targets from 1-mm to 200-μm radii (Kumavor, et al., 2011), minimizing dominant signal from microvasculatures at the higher frequency range, i.e., > 10MHz. The data recording was also extended for 10 min to validate the expectable range of pulse-to-pulse laser energy fluctuation.

Fig. S1a presents the full-width-half-maximum (FWHM) measurements were 473.0 µm, 477.8 µm, and 482.6 µm (479.5 ± 2.7 µm in average) in axial direction, and 448.5 µm, 467.6 µm, and 496.2 µm (470.8 ± 24.0 µm in average) in lateral direction at 20 mm, 25 mm, and 30 mm depths, respectively. We also evaluated the short- and long-term laser energy variation; Fig. S1b shows the energy fluctuation when various number of frame averaging is applied: 1, 5, 10, 20, 40 frames. The result proves that the median values were well converged to baseline: 0.10%, -0.07%, 0.06%, 0.14%, and 0.10% of fractional change from the global mean value, respectively. Also, the lower and upper adjacent values were limited well with more frame averaging: -8.69 – 8.69, -5.17 – 5.13, -4.52 – 4.86, -3.30 – 4.34, and -2.01 – 3.02 for 1-, 5-, 10-, 20-, 40-frame averaging, respectively. Note that our image reconstruction was with 40-frame averaging. *In vivo* signal fluctuation was much greater than error given by laser fluctuation (raw PA intensity sequence in Fig. 2). In the investigation on long-term laser energy variation, the fractional PA intensity change was only 0.15% between the first and last two minutes: 0-2 min: 0.00±1.27% vs. 8-10 min: 0.15±1.33 % (Fig. S1c). In addition, there was no significant photo-bleaching detected with given energy density, 3.5mJ/cm^2^ (Fig. S1d).

# Selection of region-of-interest (ROI) for the quantification of fPA VSD response.


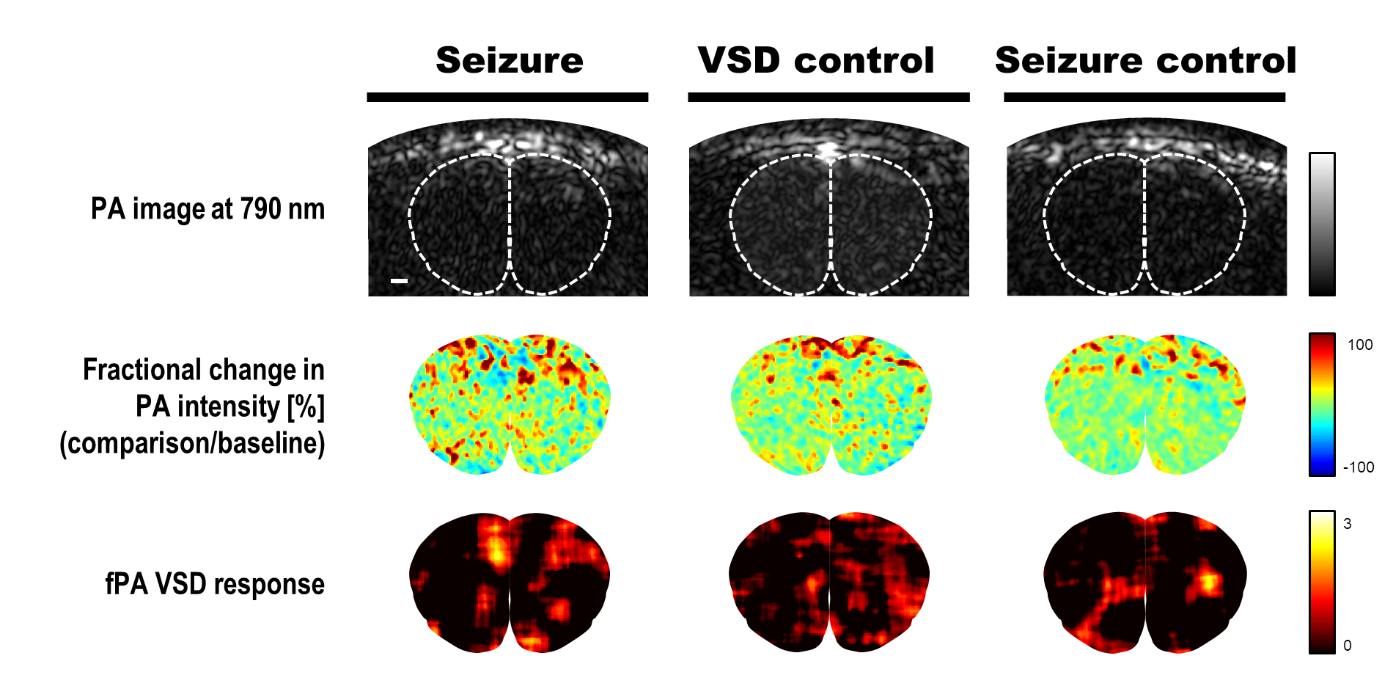


Fig. S2. Representative examples in ROI selection for fPA VSD response reconstruction and its correlation to the corresponding fractional change in PA intensity from baseline to comparison phases.

Fig. S2 presents the representative examples in ROI selection for fPA VSD response quantification based on the anatomic relationship between SSS and brain tissue. Whole brain tissue region was selected using a rat brain atlas at bregma 2.2mm as used in the *in vivo* experimental setup. The fractional change of PA intensity at 790 nm between comparison and baseline phases were also presented (second row), which demonstrate the seizure-induced CBV change. Note that the PA image was projected in last 5 min for both comparison and baseline phases.

# References

Kumavor P. K., Xu C., Aquirre A., Gamelin J., Ardeshipour Y., Tavakoli B., Zanganeh S., Alqasemi U., Yang Y., Zhu Q. (2011) Target detection and quantification using a hybrid hand-held diffuse optical tomography and photoacoustic tomography system, *J. Biomed. Opt.* **16**(4), 046010. doi: 10.1117/1.3563534.

Sun J. M. and Gerstman B. S. (1999) Photoacoustic generation for a spherical absorber with impedance mismatch with the surrounding media, *Phys. Rev. E* **59**, 5772. doi: 10.1103/PhysRevE.59.5772

Zhang M. Q., Zhou L., Deng Q. F., Xie Y. Y., Xiao T. Q., Ultra-high-resolution 3D digitalized imaging of the cerebral angioarchitecture in rats using synchrotron radiation, *Sci. Rep.* **5**, 14982. doi: 10.1038/srep14982.
